# Diagnostic evaluation of primary cervical adenopathies in a developing country

**Published:** 2011-12-06

**Authors:** Adesuwa Noma Olu-Eddo, Caroline Edijana Omoti

**Affiliations:** 1 Departments of Pathology University of Benin Teaching Hospital, Benin City, Nigeria; 2 Departments of Haematology University of Benin Teaching Hospital, Benin City, Nigeria

**Keywords:** Cervical lymphadenopathy, tuberculosis, lymphoma, metastatic cancer

## Abstract

**Introduction:**

To review the pathology of lymph node biopsies removed from patients with primary cervical lymphadenopathy.

**Methods:**

A 20 (1987-2006) year retrospective study of all patients who had lymph node biopsy; in the Department of Pathology and Haematology, University of Benin Teaching Hospital, Benin City, Nigeria.

**Results:**

Of 357 lymph node biopsies accessioned, 68 (19.0%) cases were in children. Granulomatous diseases constituted 128 (35.9%) cases. Tuberculosis (Tb) was the single commonest cause of cervical lymphadenopathy constituting 125 (35.0%) cases and also the commonest cause of cervical lymphadenopathy below the age of 45 years. Tuberculosis (TB) lymphadenopathy occurred predominantly in male children and young female adults. TB lymphadenopathy was rare above the age of 45 years. Neoplastic diseases constituted 173 (48.5%) cases. Of these, lymphoma predominated comprising 93 (26.1%) cases. These included 37 (10.4%) and 56 (15.7%) cases of Hodgkin?s lymphoma and non Hodgkin?s lymphoma respectively. Hodgkin?s lymphoma occurred most commonly in young male adults. Metastatic tumours constituted 80 (22.4%) cases and was the predominant cause of cervical lymphadenopathy above the age of 45 years. Non specific reactive lymphadenitis constituted 56 (15.7%) cases.

**Conclusion:**

Chronic lymphadenopathy in our environment has a high incidence of tuberculosis. We recommend urgent lymph node biopsy in significantly enlarged nodes not responding to treatment.

## Introduction

Cervical lymphadenopathy has been documented worldwide as the most common type of peripheral lymphadenopathy [1–3]. While tuberculosis and other infectious aetiology are reported as the predominant causes of chronic cervical lymph node enlargement in Nigeria and other parts of the tropics [2–4], malignancy is documented as the major cause of lymph node enlargement in the developed countries [5]. Recently, reports from Asia indicate a high incidence of Kikuchi lymphadenitis [6]. However, the overall high incidence of HIV infection brings a new spectrum into the differential diagnosis in certain patients [7].

Although several studies on peripheral lymphadenopathy have been done in various parts of Nigeria [2,3], there is marked paucity of information on chronic cervical lymphadenopathy as a specific entity. Chronically enlarged cervical lymph nodes therefore continue to pose a diagnostic dilemma to the Physicians and Surgeons.

In a developing country such as Nigeria, with the incessant problem of late presentation and referral, it is essential for the attending physician to have thorough knowledge of the demographic pattern of chronic cervical lymph node disorders. This will enhance prompt diagnosis and institution of definitive treatment protocols.

This study aims at defining the pattern of chronic primary cervical lymphadenopathy in children and adult patients seen in the University of Benin Teaching Hospital, Benin City, Nigeria. It is believed that information derived from this study will be of immense value to the attending physician and also form a baseline data for future research.

## Methods

All cases of lymph node biopsies received at Department of Pathology, University of Benin Teaching Hospital, Benin City, Nigeria from January 1^st^, 1987 ? December 31^st^, 2006 were reviewed. The cases of primary chronic cervical lymphadenopathy formed the focus of this retrospective study.

Clinicodemographic data regarding age, sex, anatomical site of nodal biopsy and clinical information were obtained from request cards and case files. Slides were retrieved from the archives of the Department of Pathology. Where necessary, new slides were made from formalin fixed, paraffin embedded blocks and stained with haematoxylin and eosin stains. Special stains including Ziehl Neelsen, Giemsa and Gomori?s methenamine silver were done where indicated.

Metastatic lymph nodes associated with evidence of primaries elsewhere in the body were excluded from the study.

## Results

Of the 617 lymph node biopsies reviewed during the 20 year period (1987-2006) of study, 357 biopsies were received from the cervical group of lymph nodes constituting 57.9% of all lymph nodes biopsies encountered in the Department. Analysis of the data on these 357 patients showed that while 289 (81%) patients were adults, 68 (19.0%) patients were children. There were 214 males (59.9%) and 143 females (40.1%) with a male to female ratio of 1.5: 1.

**Figure 1:**
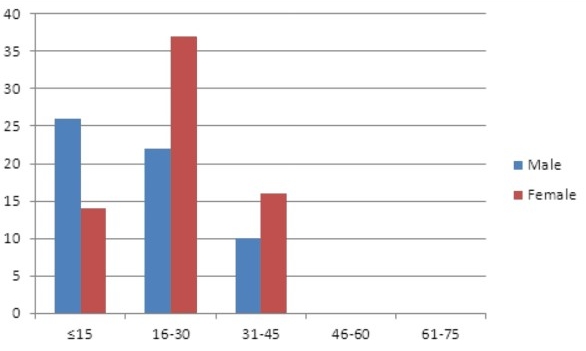
Age and sex distribution of patients with tuberculous cervical lymphadenitis

Table 1 shows the histological diagnosis of cervical lymphadenopathy. Granulomatous diseases constituted 128 (35.9%) cases. Tuberculosis (Tb) was the single commonest cause of cervical lymphadenopathy constituting 125 (35.0%) cases. Tuberculosis was also the commonest cause of cervical lymphadenopathy below the age of 45 years. The age range of patients with tuberculous lymphadenitis was 2-38 years with a peak age in the 2^nd^ decade. Overall, the male to female ratio for tuberculous lymphadenitis was 1: 1.15. However tuberculous cervical lymphadenitis occurred predominantly in male children and young adult females (Figure 1). A marked decline in the incidence of tuberculous cervical lymphadenitis was observed after the third decade with 79.2% of cases occurring before the age of 30 years. No case of tuberculous cervical lymphadenitis was seen above the age of 45 years (Table 1).

**Table 1:** Age distribution of the histological types of cervical lymphadenitis

Histological Diagnosis	Number of cases and age group (Years)						

	0-15	16-30	31-45	46-60	61-75	Total	%
**Granulomatous Diseases**							
Tuberculosis	40	59	26	-	-	125	35.0
Sarcoidosis	1	1	-	-	-	2	0.6
Cat Scratch	0	1	-	-	-	1	0.3
							
**Neoplastic diseases**							

Non Hodgkin's lymphoma	16	15	11	9	5	56	15.7
Hodgkin's lymphoma	2	23	12	-	-	37	10.4
Metastatic cancer	2	16	22	16	24	80	22.4
							
**Non specific reactive hyperplasia**							

Follicular hyperplasia	1	8	6	3	1	19	5.3
Chronic non specific lymphadenitis	5	4	4	3	2	18	5.0
Sinus histiocytosis	1	3	3	2	2	11	3.1
Dermatopathic lymphadenopathy	-	3	3	1	1	8	2.2
**Total**	**68**	**133**	**87**	**34**	**35**	**357**	**100.0**

**Figure 2:**
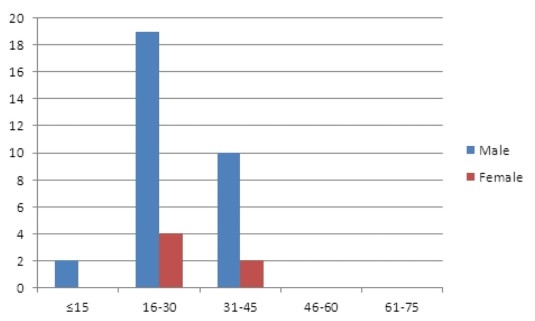
Age and sex distribution of patients with Hodgkin's lymphoma

**Figure 3:**
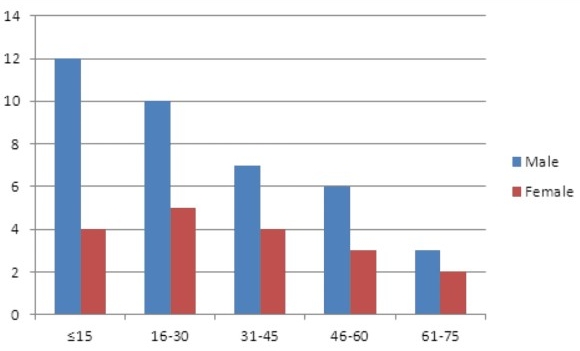
Age and sex distribution of patients with non Hodgkin's lymphoma

Neoplastic diseases constituted 173 (48.5%) cases. Of these, lymphoma predominated comprising 93 (26.0%) cases. These included 37 (10.4%) and 56 (15.7%) cases of Hodgkin?s lymphoma and Non Hodgkin?s lymphoma (NHL) respectively. The age range of patients with Hodgkin?s lymphoma was 6-44 years. However, Hodgkin?s lymphoma occurred most commonly in young male adults (Figure 2) with a male to female ratio of 4:1. NHL showed a wider age range of 3-74 years and a male predominance with a male to female ratio of 2.1:1 (Figure 3).

Table 2 shows the histological types of metastatic cancer encountered in this study. Metastatic tumours constituted 80 (22.4%) cases and was the predominant cause of cervical lymphadenopathy above the age of 45 years. Only 2 (0.6%) of these cases were encountered in children. Non specific reactive lymphadenitis constituted 56 (15.7%) cases. The age distribution of the histologic types of non specific reactive lymphadenitis are shown in Table 1.

**Table 2:** Age and sex distribution of histopathological types of metastatic lymph nodes

Histological diagnosis	Total	Male	Female	Male/Female	Age range (years)	Mean age (Years)
Squamous cell carcinoma	56	32	24	1.3:1	10-78	48.0
Adenocarcinoma	16	13	3	4.3:1	31-70	51.4
Malignant melanoma	6	3	3	1:1	58-71	65.2
Kaposi sarcoma	1	1	0		45	
Medullary carcinoma of thyroid	1	0	1		53	
**Total**	**80**	**48**	**31**	**1.5:1**	**20-78**	**50.3**

## Discussion

Chronic cervical lymphadenopathy is a common clinical problem frequently requiring surgical biopsy. We retrospectively studied 357 patients with surgically removed cervical lymph nodes over a 20-year period (1984-2003).

In our series, cervical lymphadenopathy resulting from granulomatous disease made up 35.9%. Tuberculosis constituted 35.0% of cases and was the main cause of chronic granulomatous infections presumably due to the overall high prevalence of the disease in the country and subregion [2,3]. Moreover, tuberculosis has previously been documented in several reports as the commonest cause of cervical lymphadenopathy in the tropics [2–4,8,9]. Furthermore, peripheral tuberculous lymphadenopathy has been reported as the commonest form of extra pulmonary tuberculosis [1]. Consistent with previous reports from Nigeria and other parts of the tropics [2–4], most of the patients with primary cervical lymphadenitis were male children, and young female adults. This may be attributed to the fact that tuberculous lymphadenitis is an early post primary complication.

More so it has been observed that, in countries with a high prevalence of tuberculosis, people are exposed more intensively, on average, and show tuberculosis at an earlier age. Lymphoma was the 2nd commonest specific cause of lymphadenopathy constituting 26.0% of cases. These included 37 (10.4%) and 56 (15.7%) cases of Hodgkin?s lymphoma and Non Hodgkin?s lymphoma (NHL) respectively. While Hodgkin?s lymphoma occurred predominantly in young adult males with paucity of cases above the age of 45 years, there was a relatively wider spread of cases of non Hodgkin?s lymphoma with ages ranging from 3 -74 years. In keeping with other previous reports from the tropics [2–4]. NHL was however more common in children and young adults with 55.4% of cases of NHL occurring below the age of 30 years.

In agreement with most previous reports from Nigeria and other parts of the tropics [2–4], metastatic carcinoma (22.4%) was the predominant cause of chronic cervical lymphadenopathy in patients above 45 years with a striking rarity in children.

Squamous cell carcinoma was the commonest type of metastatic lesion in the cervical lymph nodes constituting 70.9% of cases of metastatic cancer. Consistent with most previous reports [2], the predominant primary lesion was a squamous cell carcinoma of the head and neck region. This has diagnostic implications as success in the control of cancer is inversely proportional to the interval between onset and diagnosis. Thus there is a dire need for prompt diagnosis and institution of treatment protocols.

Non specific reactive lymphadenopathy has been documented as a common cause of peripheral lymph node enlargement in the tropics with rates ranging from 15-22% in adults [2,3,8,10] and 20.6- 41.0% in children [11,12]. In this study, non specific reactive lymphadenopathy constituted 56 (15.7%) cases. It is worth noting that only 7 (1.96%) of these cases were in children. Non specific reactive cervical lymphadenitis seems to be relatively uncommon in children. Moreover, in childhood, a predominance of inguinal lymph node enlargement attributed to the tendency of the locals to move around bare footed has been documented [6,8,10].

The association of Human immunodeficiency virus (HIV) infection with tuberculosis and tumours has been widely documented [7]. In this study, HIV positivity was observed in 7(10.6%) of the 66 patients tested. These included 6 cases of tuberculous lymphadenitis and a case of Kaposi sarcoma.

Fine-needle aspiration biopsy has been reported by several authors as a reliable, safe, less invasive and relatively cheap procedure and has been advocated as an alternative to surgical biopsy in the diagnosis of lymph node disorders [13]. While lymph node biopsy is still the major diagnostic procedure in this centre with a yield of 95%, comparable to 75-100% reported by other authors [2,3,8,10], it is fraught with incessant delays in diagnosis resulting from inadequate theatre facilities with long waiting lists and limited manpower.

## Conclusion

In this study, primary chronic cervical lymphadenopathy was found to have a high incidence of tuberculosis. The incidence of a specific pathology in more than half the cervical lymph nodes examined justifies the need for urgent investigation of significantly enlarged nodes not responding to treatment.
